# Controlled labelling of tracer antibodies for time-resolved fluorescence-based immunoassays

**DOI:** 10.1038/s41598-024-69294-7

**Published:** 2024-08-05

**Authors:** Anastasiia Kushnarova-Vakal, Rami Aalto, Tuomas Huovinen, Saara Wittfooth, Urpo Lamminmäki

**Affiliations:** 1 Department of Life Technologies, University of Turku, 20520 Turku, Finland; 2 InFLAMES Research Flagship, University of Turku, 20014 Turku, Finland; 3 Tyks Laboratories, Clinical Chemistry, Turku University Hospital, 20521 Turku, Finland

**Keywords:** Biotechnology, Chemical biology

## Abstract

Tracer antibodies, which are labelled with fluorescent or other type of reporter molecules, are widely employed in diagnostic immunoassays. Time-resolved fluorescence immunoassay (TRFIA), recognized as one of the most sensitive immunoassay techniques, utilizes tracers labelled with lanthanide ion (Ln) chelates. The conventional approach for conjugating isothiocyanate (ITC) Ln-chelates to antibodies involves random chemical targeting of the primary amino group of Lys residues, requiring typically overnight exposure to an elevated pH of 9–9.3 and leading to heterogeneity. Moreover, efforts to enhance the sensitivity of the assays by introducing a higher number of Ln-chelates per tracer antibody are associated with an elevated risk of targeting critical amino acid residues in the binding site, compromising the binding properties of the antibody. Herein, we report a method to precisely label recombinant antibodies with a defined number of Ln-chelates in a well-controlled manner by employing the SpyTag/SpyCatcher protein ligation technology. We demonstrate the functionality of the method with a full-length recombinant antibody (IgG) as well as an antibody fragment by producing site-specifically labelled antibodies for TRFIA for cardiac troponin I (cTnI) detection with a significant improvement in assay sensitivity compared to that with conventionally labelled tracer antibodies. Overall, our data clearly illustrates the benefits of the site-specific labelling strategy for generating high-performing tracer antibodies for TRF immunoassays.

## Introduction

Distinguished by their remarkable capability to tightly bind to specific molecules of interest, antibodies play a crucial role in various applications. Of these, immunoassays stand out as one of the most essential and widely employed techniques. Monoclonal antibodies produced using the conventional hybridoma technology still dominate in immunoassays. However, the advantages shown by antibodies produced in recombinant hosts, such as mammalian cell lines or bacteria, have led to increased use of the recombinant antibodies. One of the main advantages of recombinant antibodies relates to the possibility to tailor their properties by means of genetic engineering. For example, mutations can be targeted to the variable domain regions of antibodies to modulate their binding properties, such as affinity and specificity. In addition, various peptide or protein domains can be fused to the termini of antibody polypeptides, allowing improved immobilization or detection^1,2^. Moreover, recombinantly produced antibody fragments of smaller sizes, such as single chain Fv (scFv) and fragment antigen binding (Fab) may improve diagnostic assay performance^3,4^.

Time-resolved fluorometry (TRF) is one of the most sensitive reporter technologies for immunoassays. TRF immunoassays (TRFIA) make use of the capacity of some rare earth metal ions, lanthanides (Ln), such as europium(III) and terbium(III)^5^. Ln can form highly fluorescent chelates that possess unique photophysical properties including high quantum yield, large Stokes shifts (> 150 nm), narrow-banded emission lines (< 10 nm at half-maximum), and very long lifetime of the excited state (0.1–1.0 ms). Depending on the type of the Ln chelate used, the TRF signal is measured either directly from the antibody-bound self-fluorescent chelate or from the solution after low pH-induced detachment of the Ln ion from non-fluorescent carrier chelate. The latter technique is known as the dissociation-enhanced lanthanide fluorescence immunoassay (DELFIA)^6–8^. Owing to the long fluorescence life-time, the emission signal of Ln-chelates can be measured in a time-gated manner. This efficiently omits the short-lived background fluorescence from the other assay components^9^ and has a significant contribution to the high sensitivity of TRFIA. A remaining factor potentially constraining the sensitivity of TRFIA is a limited specific activity of the tracer, which refers to the signal strength from each labelled antibody. To address this, there is often interest in increasing the number of chelate labels per tracer, thereby enhancing specific activity and improving assay sensitivity.

Like most approaches for labelling proteins with small chemical compounds such as biotin or fluorophores, conjugation of Ln chelates to antibodies involves chemical targeting of suitable functional groups of the surface exposed amino acids of the antibodies. Generally, such labelling reactions are performed via either epsilon amine in Lys residue by using reagents with, e.g., *N*-hydroxysuccinimide (NHS) ester or isothiocyanate (ITC), or via sulfhydryl group of cysteine with, e.g., maleimide or iodoacetamide^10^. In the case of Ln-chelates, ITC-activated chelates are typically used: a very electrophilic carbon atom of ITC efficiently reacts with a free deprotonated side chain amino group of lysine or, to a lesser extent, with the N-terminus to form a stable, covalent thiourea bond. Chemical labelling of the antibodies leads to a random targeting of lysines within the antibody, potentially affecting the binding site residues and thus the antigen binding capability. Furthermore, in order to obtain optimal ITC labelling degree the antibodies have to be subjected to an increased pH level of > 9 for several hours^11–13^. This may lead to denaturation and aggregation especially in less stable antibody types, such as scFvs^14,15^.

A highly efficient protein ligation platform based on a *Streptococcus pyogenes* derived^16^ protein-peptide pair has been described by Zakeri et al.^17^. The platform comprises of a 15.5-kDa SpyCatcher domain and 16 amino acids SpyTag peptide, capable of rapidly reacting with each other to form an irreversible isopeptide bond across a wide range of temperatures, buffers, and pH values^18,19^. Both SpyCatcher and SpyTag can be expressed as a fusion with a protein of interest, enabling covalent glueing of the fusion proteins upon mixing. Moreover, the SpyTag peptide can also be chemically synthesized, allowing its convenient chemical modification as a mere peptide. This versatile protein ligation technique has been successfully implemented in numerous different applications^20–25^, and it is well suited to be applied in the context of recombinant antibodies.

Herein, we present a gentle and straightforward method for site-specific labelling of antibodies for TRFIA using the SpyTag/SpyCatcher protein ligation system. In this method, a synthesized Lys-rich peptide containing the SpyTag sequence is first chemically conjugated to saturation with Eu-chelates and then attached to the antibody fusions containing the SpyCatcher domain. We demonstrate the effectiveness of the technique by labelling two types of antibodies: a bacterially expressed scFv and a mammalian cell expressed intact IgG to produce site-specifically labelled tracer antibodies for TRFIA. The performance of the labelled antibodies is presented in a TRFIA for cardiac troponin I (cTnI), a biomarker of myocardial injury that requires high assay sensitivity^26,27^.

## Results

### Production and purification of scFv-SpyCatcher003 antibody

**Figure 1:**
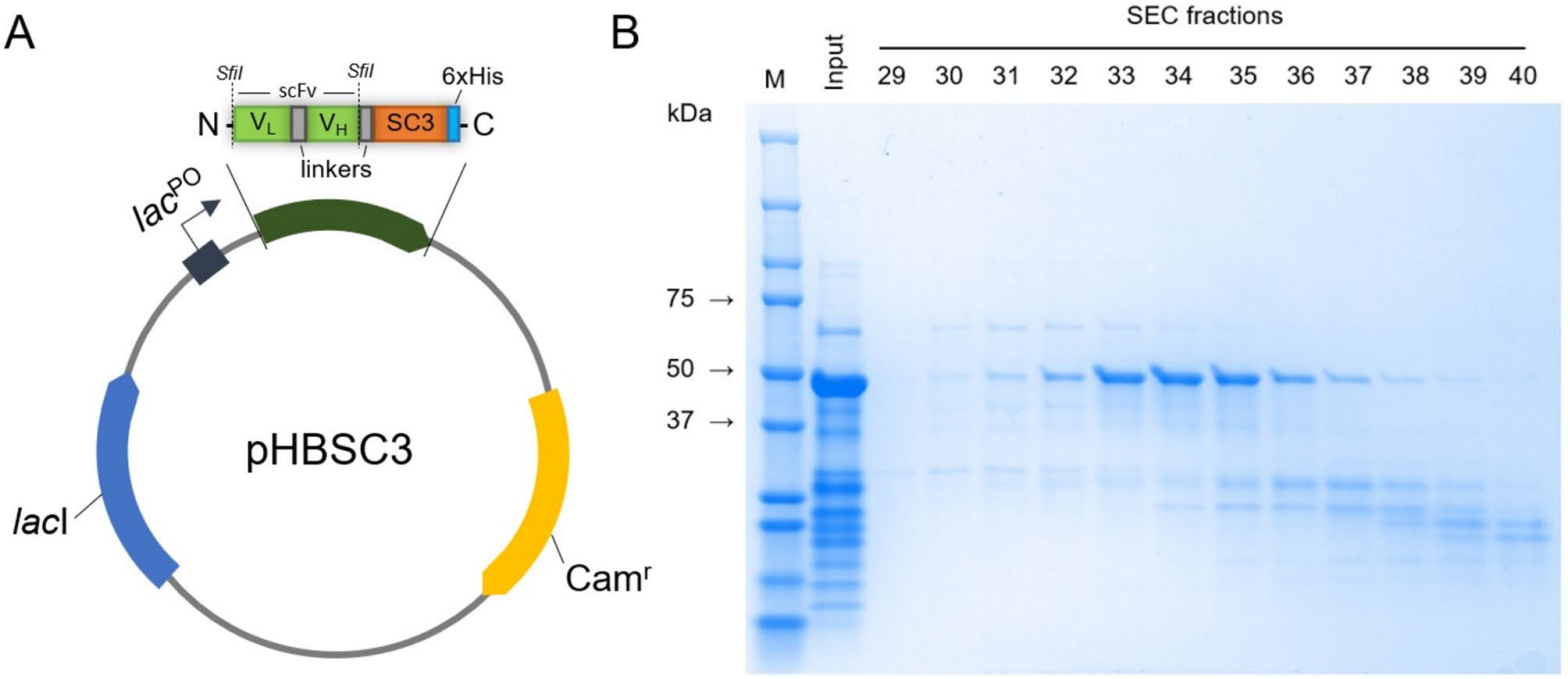
Production of 11N11 scFv-SpyCatcher003 antibody in *E. coli*. (**A**) Schematic representation of the recombinant pHBSC3 expression vector. SC3, SpyCatcher003; variable light chain (V_L_) and variable heavy chain (V_H_) connected via a short linker form a single-chain variable fragment (scFv). (**B**) SDS-PAGE analysis of SEC-purified 11N11 scFv-SpyCatcher003 antibody. The input corresponds to the Ni-NTA elution sample. M, molecular weight markers.

To generate an 11N11 scFv-SpyCatcher003 antibody, a bacterial expression vector pHBSC3 with a C-terminally fused SpyCatcher003^19^ followed by a hexa histidine-tag (Fig. 1A) was used. The scFv-SpyCatcher003 construct was expressed into the periplasm of *E. coli* and purified by Ni-NTA affinity chromatography and size exclusion chromatography (SEC). After SEC, the correct molecular size of 11N11 scFv-SpyCatcher003 antibody (41.8 kDa) was verified with SDS-PAGE (Fig. 1B) analysis. Pooling of the fractions 32–36 yielded 1.8 mg of purified antibody.

### Production and purification of IgG and IgG-SpyCatcher003 antibodies

**Figure 2:**
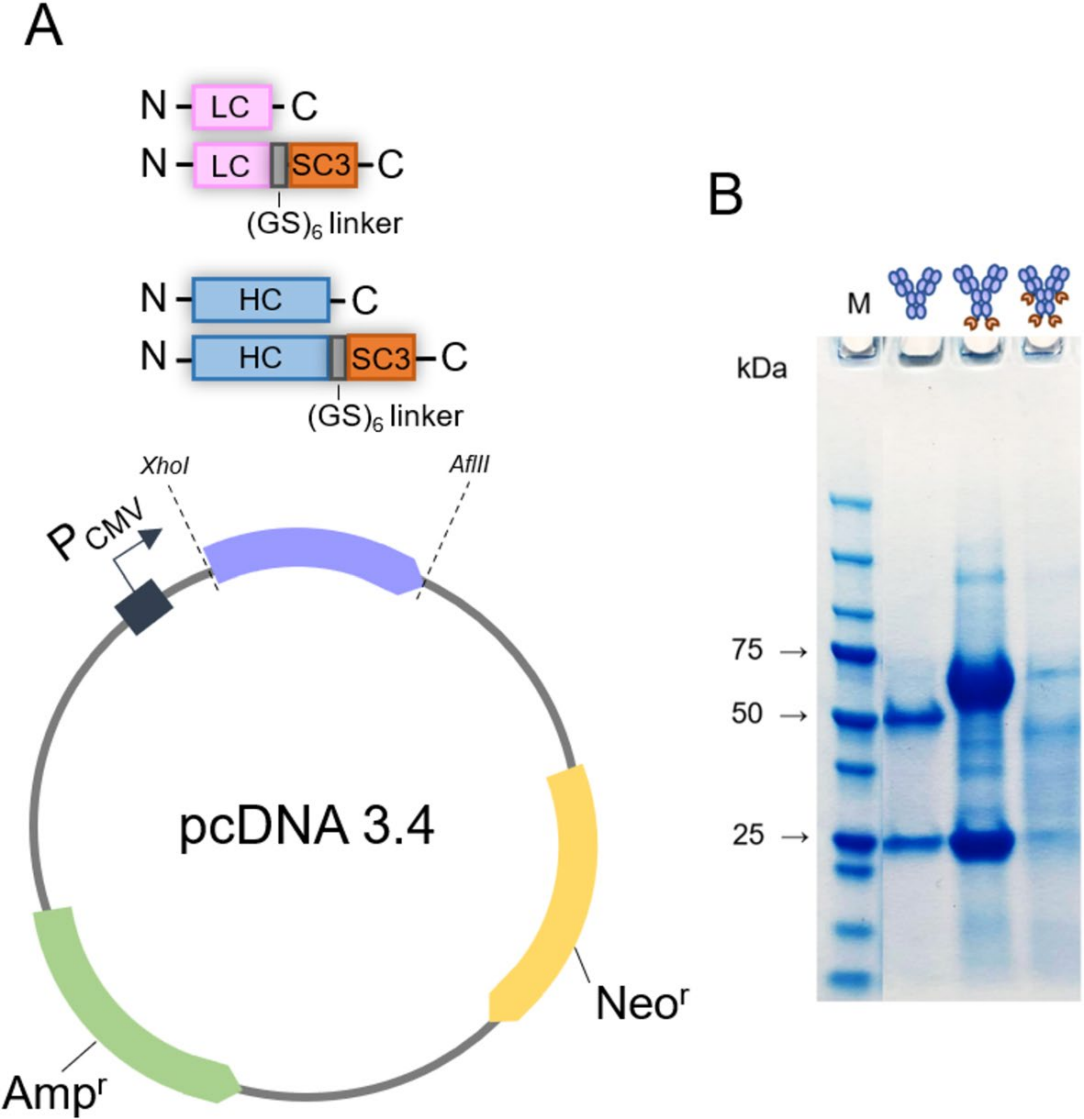
Transient expression of IgG-SpyCatcher003 antibodies in mammalian cells. (**A**) Schematic representation of the recombinant pcDNA 3.4 expression vector. LC, light chain; HC, heavy chain; SC3, SpyCatcher003. (**B**) SDS-PAGE analysis of protein A purified IgG and IgG-SpyCatcher003 antibodies. M, molecular weight markers. A full image of the SDS-PAGE gel is provided in Supplementary Information.

The 11N11 IgG-SpyCatcher003 antibodies were produced in ExpiCHO cells which were co-transfected with the light (LC) and heavy (HC) chain encoding pcDNA3.4 expression vectors. Two different IgG-SpyCatcher fusion constructs were designed: one having SpyCatcher003 fused to the C-termini of both the light and heavy chain of IgG and another one having it fused only to the heavy chain (Fig. 2A). Thus the corresponding expression products were expected to have either four or two SpyCatcher003 domains per antibody, respectively. Additionally, we produced the intact IgG lacking the SpyCatcher003 domain.

The expressed 11N11 IgG and IgG-SpyCatcher003 antibodies were purified using a protein A resin, which efficiently captured the antibody also when fused to SpyCatcher003. The protein yields after protein A purification were 0.9 and 1.7 mg/l of transfected culture medium for the unmodified control IgG and the IgG with SpyCatcher003 on both light and heavy chains, respectively. Surprisingly, the IgG construct with a SpyCatcher003 only on the heavy chain resulted in an 80- to 150-fold higher yield than the other constructs equivalent to 135.1 mg/l of transfected culture medium.

The purified 11N11 full-length antibodies were analysed in reducing SDS-PAGE (Fig. 2B), and correct bands (light chain: 24.2 kDa; light chain-SpyCatcher003: 37.2 kDa; heavy chain: 49.0 kDa; heavy chain-SpyCatcher003: 62.0 kDa) for the engineered heavy and light chains were observed. The 11N11 IgG with a SpyCatcher003 on the heavy chain was selected to be used for assessing the proof-of-concept labelling strategy.

### Design, labelling and purification of the SpyTag003-polylysine peptide

**Figure 3:**
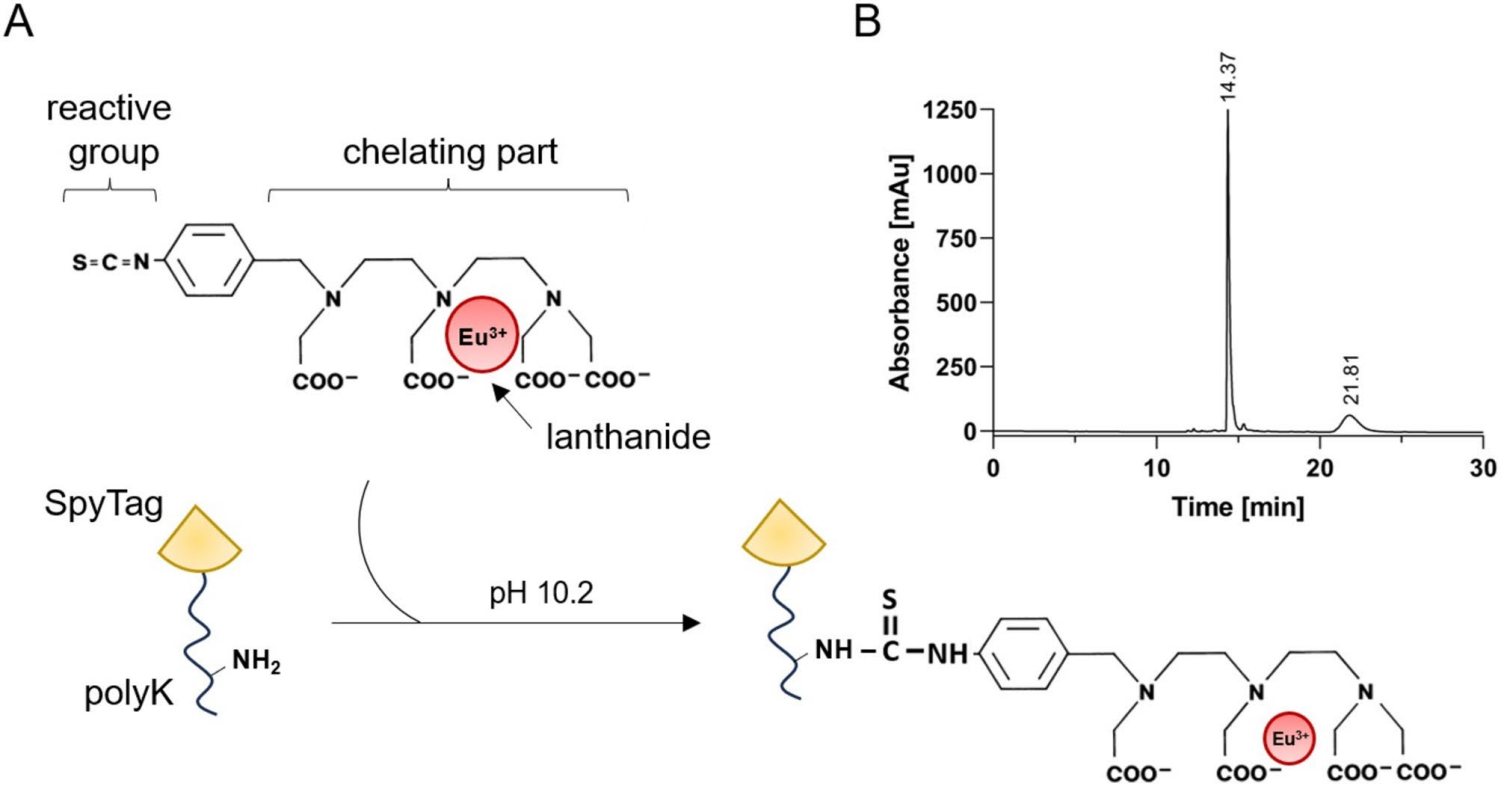
Labelling and purification of SpyTag003-polyK peptide. (**A**) Schematic representation of the reaction between SpyTag003-polyK peptide and Eu(III)-N1-ITC chelate. (**B**) HPLC chromatogram of the Eu-labelled SpyTag003-polyK peptide.

The SpyTag003 sequence-containing peptide was designed to have multiple free side chain amino groups available for the reaction with the isothiocyanate (ITC) group of Eu(III)-N1-ITC chelate (Fig. 3A). The prototype 41 amino acids (4.9 kDa) peptide, SpyTag003-polyK, consists of the N-terminal SpyTag003 sequence followed by a short linker (Gly–Gly–Gly–Gly–Ser) and twenty lysine residues (polyK). The N-terminal end of the peptide was acetylated to avoid ITC reactivity with N-terminus. Various optimization steps, such as adjusting the pH of the buffer and testing different reaction times and chelate concentrations, were conducted in order to obtain the highest possible labelling degree (data not shown). The maximum degree of labelling (8.6 Eu-chelates/peptide) was achieved by using a 75-fold molar excess of Eu-chelate at pH 10.2 overnight at RT (Fig. 3A). The Eu-labelled SpyTag003-polyK peptide was then purified with HPLC (Fig. 3B). Two distinct peaks were observed at 280 nm, with the peaks at 14.37 and 21.81 min representing the excess of Eu-chelate and the labelled peptide, respectively. The second peak was collected, evaporated, and subsequently used for coupling with the antibody-SpyCatcher003 fusions.

### Coupling of antibody-SpyCatcher003 fusions to SpyTag003-containing peptides

**Figure 4:**
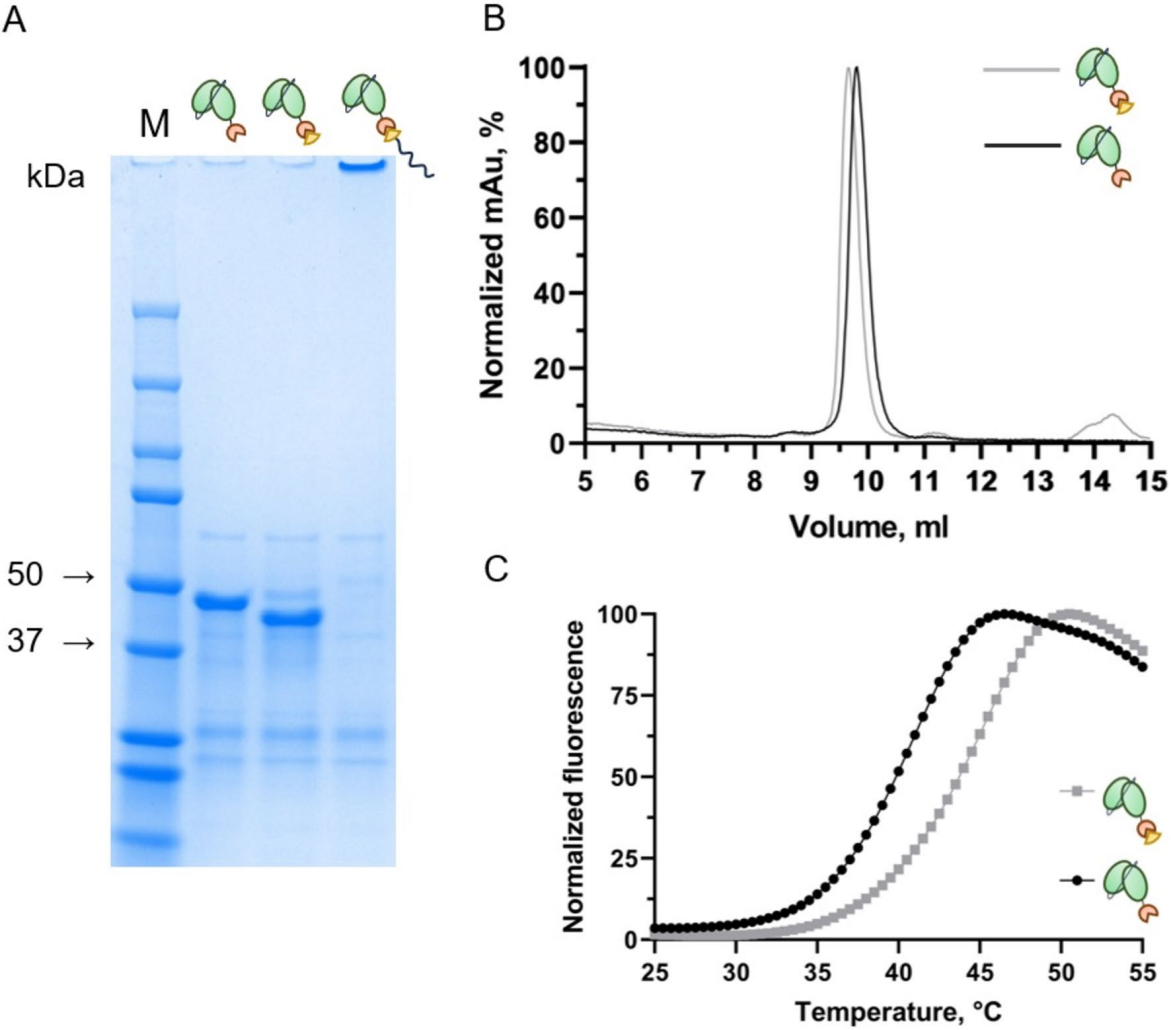
Coupling of 11N11 scFv-SpyCatcher003 antibody with SpyTag003 and SpyTag003-polyK peptides. (**A**) SDS-PAGE analysis of 11N11 scFv-SpyCatcher003 antibody coupled with SpyTag003 and SpyTag003-polyK peptides. (**B**) SEC profiles of 11N11 scFv-SpyCatcher003 before (black) and after (grey) coupling with SpyTag003 peptide. (**C**) Differential scanning fluorimetry (DSF) curve of the 11N11 scFv-SpyCatcher003 before (black) and after (grey) coupling with SpyTag003 peptide.

**Figure 5:**
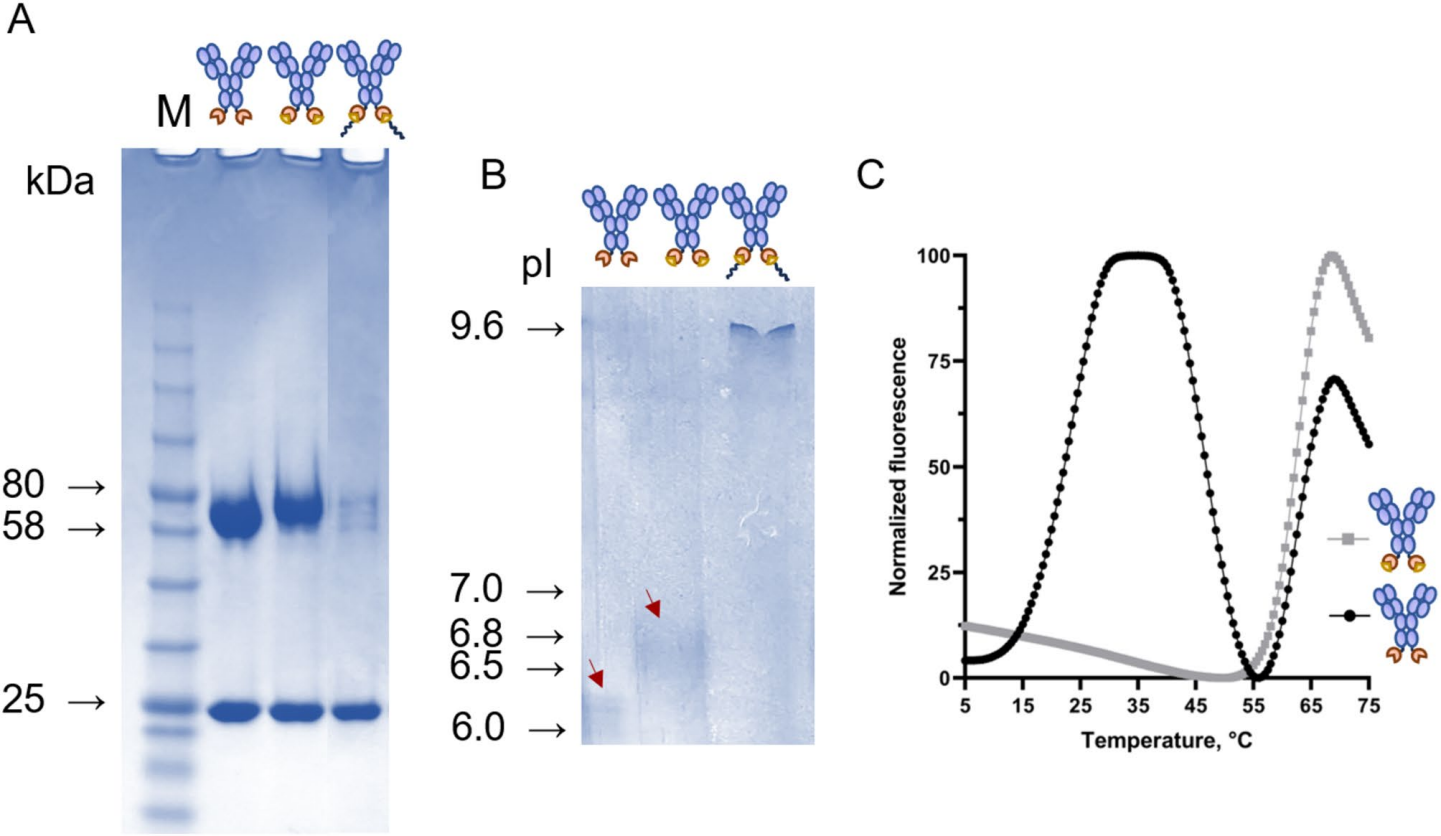
Analysis of 11N11 IgG-SpyCatcher003 antibody complexation with the SpyTag003 and SpyTag003-polyK peptides. (**A**) SDS-PAGE analysis of 11N11 IgG-SpyCatcher003 antibody (lane 2) and as coupled with SpyTag003 peptide (lane 3) and SpyTag003-polyK peptide (lane 4). A full image of the SDS-PAGE gel is provided in Supplementary Information. (**B**) Isoelectric focusing (IEF) of 11N11 IgG-SpyCatcher003 antibody (lane 1) and as complex with SpyTag003 peptide (lane 2) and with SpyTag003-polyK peptide (lane 3). A full image of the IEF gel is provided in Supplementary Information. (**C**) DSF curve of the 11N11 IgG-SpyCatcher003 before (black) and after (grey) coupling with SpyTag003 peptide.

To verify the reactivity of 11N11 scFv-SpyCatcher003 and IgG-SpyCatcher003 antibodies with SpyTag003-containing peptides (mere SpyTag003 and SpyTag003-polyK), the antibodies were mixed with peptides in 1:1 and 1:2 ratios, respectively. The formation of isopeptide bond was confirmed by heat treatment and reducing SDS-PAGE analysis (Figs. 4A, 5A). Interestingly, the 11N11 scFv-SpyCatcher003 antibody (41.8 kDa) after complexation with SpyTag003 migrated counterintuitively faster appearing to be of lower molecular weight than the noncomplexed scFv-SpyCatcher003. To further investigate the difference in sizes of 11N11 scFv-SpyCatcher003 (41.8 kDa) and scFv-SpyCatcher003/SpyTag003 (43.8 kDa), we analysed the samples with SEC. Based on the layout chromatograms (Fig. 4B), scFv-SpyCatcher003 is indeed a larger molecule after complexation with SpyTag003 as the peak for scFv-SpyCatcher003/SpyTag003 appears earlier, at 9.66 ml, while scFv-SpyCatcher003 comes at 9.79 ml.

The heavy chain of 11N11 IgG-SpyCatcher003 antibody ran as a higher molecular weight band after the complexation (Fig. 5A, lane 3). The band shift for the heavy chain was in accordance with our construct design: 62.0 kDa for heavy chain-SpyCatcher003 and 64.0 kDa for heavy chain-SpyCatcher003/SpyTag003. However, the expected band of heavy chain-SpyCatcher003/SpyTag003-polyK (66.9 kDa) did not appear on the gel (Fig. 5A, lane 4). We hypothesized that it was due to the highly positive charge of the polyK tail of the peptide: treatment with the negatively charged ionic detergent SDS for PAGE does not seem to be efficient enough to overcome the high positive charge of polyK tail, thus, preventing the conjugate to move towards anode and hence entering the PAGE-gel. The theoretical calculation and IEF-based analysis (Fig. 5B) of the pI value showed that the pI of IgG-SpyCatcher003/SpyTag003-polyK (theoretical 8.5, 9.4–9.5 on the gel) is significantly higher compared to that of IgG-SpyCatcher003 (theoretical pI 5.8, 6.1–6.2 on the gel) and IgG-SpyCatcher003/SpyTag003 (theoretical pI 6.0, 6.4–6.5 on the gel). Unsurprisingly, the scFv-SpyCatcher003/SpyTag003-polyK antibody could not enter the gel either and the expected band of 46.7 kDa was not seen on the gel (Fig. 4A, lane 4).

We also studied by differential scanning fluorometry (DSF) whether the thermal stability of the 11N11 antibody-SpyCatcher003 fusions is affected by the complexation with SpyTag003 peptide. The observed melting temperature (Tm) for the 11N11 scFv-SpyCatcher003 in the absence of SpyTag003 was as low as 39.7 °C, and elevated slightly to 43.5 °C, upon complexation with SpyTag003 (Fig. 4C). The Tm of the IgG-SpyCatcher003/SpyTag003 antibody was 62.2 °C, while IgG-SpyCatcher003 alone shows a biphasic melting pattern likely due to instability of the SpyCatcher003 domain in the absence of SpyTag003 (Fig. 5C).

### Labelling antibodies for TRFIA with Eu-labelled SpyTag003-polyK and Eu(III)-N1-ITC chelate

To connect antibodies with Ln chelates, 11N11 scFv-SpyCatcher003 and IgG-SpyCatcher003 were mixed with the Eu-labelled SpyTag003-polyK peptide in 1:1 and 1:2 ratio, respectively. After overnight incubation at 4 °C with rotation, antibodies were purified with SEC. The resulting degree of labelling (DoL) was determined to be 7.9 and 17.8 Eu per scFv and IgG, respectively.

For comparison, antibodies were also labelled in a conventional non-specific manner allowing the chelate to react directly with the antibodies. The 11N11 scFv-SpyCatcher003 was mixed either with 25-fold or 100-fold molar excess of Eu(III)-N1-ITC chelate overnight at RT followed by SEC purification that resulted in 2.3 and 7.2 Eu-chelates/scFv, respectively. Similarly, 11N11 IgG-SpyCatcher003 was mixed either with a 50-fold, 150-fold, or 300-fold molar excess of Eu(III)-N1-ITC chelate followed by overnight incubation and SEC purification resulting in products with 2.8, 8.7, and 25.7 Eu-chelates/IgG, respectively.

### Performance in TRFIA

The performance of site-specifically labelled 11N11 tracer antibodies was evaluated in TRFIA. The antibody 11N11 recognizes a C-terminal epitope at residues 160–179 of cardiac troponin I (cTnI)^28^, one of the three troponin subunits forming the cardiac ITC complex^29^. As capture antibodies, commercially available cTnI-specific antibodies 4C2 and 19C7 recognizing N-terminal and mid-fragment epitopes at residues 23–29 and 41–49, respectively^30^, were used.

**Figure 6:**
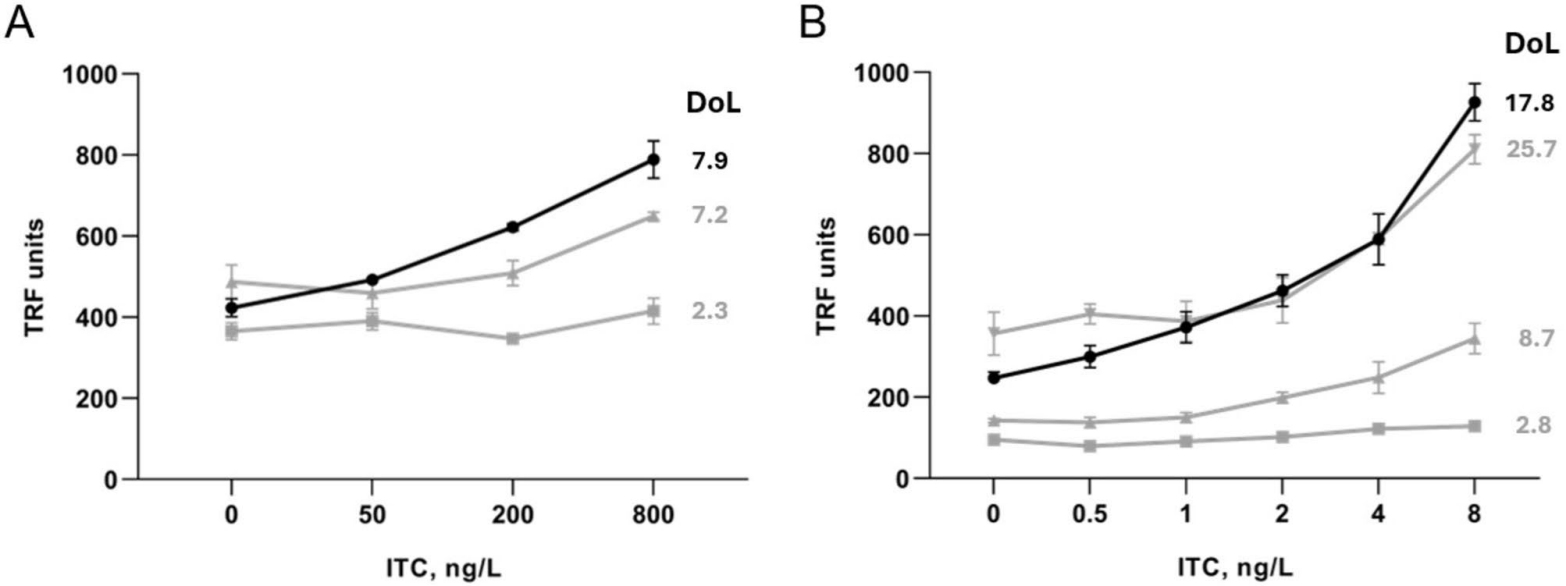
Performance evaluation for site-specifically labelled 11N11 scFv-SpyCatcher003/SpyTag003-polyK antibody in TRFIA. (**A**) Detection of the cTnI with site-specifically labelled 11N11 scFv-SpyCatcher003/SpyTag003-polyK antibody (black; 7.9 Eu-chelates/scFv) and non-specifically labelled scFv-SpyCatcher003/SpyTag003 antibodies (grey; 2.3 Eu-chelates/scFv and 7.2 Eu-chelates/scFv). (**B**) Detection of the cTnI with site-specifically labelled 11N11 IgG-SpyCatcher003/SpyTag003-polyK antibody (black; 17.8 Eu-chelates/IgG) and non-specifically labelled IgG-SpyCatcher003/SpyTag003 antibodies (grey; 2.8 Eu-chelates/IgG, 8.7 Eu-chelates/IgG and 25.7 Eu-chelates/IgG). Data represents mean ± SD (n = 3).

The sandwich-type TRFIA was initially fine-tuned for optimal immunoassay conditions with tests encompassing varying quantities of tracer antibodies, capture antibodies, and immunoassay duration. The optimal TRFIA with scFv antibody format as a tracer was a two-step immunoassay employing only 4C2 as a capture antibody, whereas the TRFIA with IgG antibody as a tracer was a one-step immunoassay with both 4C2 and 19C7 as capture antibodies. The detection limit (LoD; blank + 3SD) of the resulting immunoassay with site-specifically labelled scFv antibody as a tracer was 43.0 ng/l (Fig. 6A). The corresponding immunoassay with the conventionally labelled tracer scFv antibody with DoL of 7.2 yielded LoD of 652.2 ng/l, while another conventionally labelled scFv with DoL of 2.3 was not able to distinguish cTnI in the tested range. When the site-specifically labelled IgG antibody was employed as a tracer, the detection limit of the immunoassay was 0.16 ng/l (Fig. 6B). The use of conventionally labelled tracer IgG antibodies with DoL of 8.7 and 25.7 Eu-chelates/IgG resulted in the detection limit of 0.72 ng/l and 2.39 ng/l, respectively. The non-specifically labelled IgG with a DoL of 2.8 was not able to distinguish cTnI in the tested range.

## Discussion

Boosting the specific activity of tracer antibodies plays a pivotal role in the efforts to create increasingly sensitive immunoassays. In TRFIA, this entails maximizing the labelling degree i.e., number of Ln-chelate labels per antibody molecule. However, efforts to reach a high labelling degree can have a negative impact on the structure of the antibody compromising its functionality. The optimal number of chelates per antibody varies depending on the intricate structural features of different antibodies. According to commercial providers of Ln-chelates, the recommended range for intact IgG antibodies (molecular weight (MW) of 150 kDa) is typically 4–15 chelates per antibody. For smaller proteins like scFv antibody fragments with a MW below 30 kDa, the recommendation is 1–3 chelates per protein^31^.

Here, we report a convenient and gentle method for labelling antibodies with a high number of Ln chelates avoiding the risk of chemical modifications in the antibody’s binding site by utilizing the SpyTag/SpyCatcher protein ligation platform^17–19^ for site-specific conjugation. The SpyCatcher domain is genetically fused to the C-terminus of the antibody constructs in both studied antibody formats, scFv and IgG. This places the SpyCatcher domain and the SpyTag-linked Ln-chelate labels distant from the antigen binding site of the antibody, hereby also mitigating the risk of compromising the antibody functionality. Furthermore, our approach, in contrast to the conventional labelling with Ln-chelates, allows the conjugation of the labels to the antibody in a neutral buffer. Hence, exposure of the antibody to potentially harmful alkaline conditions with pH > 9 is avoided while the peptide, labelled separately before its conjugation to the antibody, can easily tolerate such environment.

To evaluate the performance of Eu-labelled tracer antibodies produced by the site-specific labelling approach, we compared them to tracers produced with the conventional random labelling in TRFIA. The assay targeted cTnI, a vital biomarker for early myocardial injury diagnosis^26,27^. We observed a significant improvement in immunoassay sensitivity with site-specific labelling of tracer antibodies for both antibody formats studied, IgG and scFv. In the case of IgG, the sensitivity improved by 4.5-fold compared to the most optimally conventionally labelled variant (with a labelling degree of 8.7), while for scFv, the sensitivity improved by 15-fold. The pronounced effect in the case of scFv is consistent with the limited stability and the small size of scFv. In the case of IgG, the performance of the non-specifically labelled tracer deteriorated especially when high load of the Eu-chelates was present. The level of background signal associated with the unspecific binding of the tracer correlated with the labelling degree of both specifically and non-specifically labelled IgG, limiting the sensitivity and leaving room for further optimization. In this study, a single peptide design, composed of SpyTag003 followed by a five-residue linker and twenty lysines, was used. To further enhance the assay performance, peptides with modified chemical composition and structure could be studied to reduce the non-specific binding and/or further increase the specific activity of the tracer.

In the current study, we utilized the DELFIA principle-compatible Eu-chelate. However, the concept should be equally applicable when using intrinsically fluorescent lanthanide chelates. These chelates allow for direct measurement of the fluorescence signal without the additional fluorescent enhancement step thus being particularly suitable for rapid point-of-care applications^32–34^. Unlike conventional prompt fluorophores, Ln chelates do not experience the self-quenching phenomenon. This enables the placement of multiple labels adjacent to each other without loss of fluorescence activity.

In conclusion, the present study outlines the development and implementation of site-specific labelling of antibodies for TRFIA applications. Our labelling strategy can significantly enhance assay sensitivity by maximizing the specific activity of the antibody tracer through a high number of labels per antibody, without compromising the antibody's binding function. Along with the increasing availability of high-quality diagnostic antibodies in recombinant format, our findings provide a promising approach to further push the sensitivity of TRFIA.

## Materials and methods

### Cloning, expression, and purification of scFv antibody

Expression vector pHBSC^35^ was upgraded to pHBSC3 by replacing the truncated SpyCatcher001 gene with the SpyCatcher003 gene using PCR and Gibson assembly. SpyCatcher003 gene was amplified from template pDEST14-SpyCatcher003^19^ with primers TH404 and TH405, and the pHBSC vector backbone with primers TH406 and TH407. All primer sequences can be found in Supplementary information. The Gibson reaction was transformed to *E. coli* XL1-Blue cells (Santa Clara, USA), and the final construct was verified by DNA sequencing (Macrogen, The Netherlands) from miniprep DNA (GeneJet Plasmid Miniprep Kit, Thermo Scientific, USA).

The gene of 11N11 cardiac troponin I-binding single-chain variable fragment antibody (scFv) was cloned to pHBSC3 expression vector with *SfiI* restriction sites. The obtained plasmid was transformed into electrocompetent XL1-Blue *E. coli* cells, and transformant cells were grown on LA-agar plates supplemented with 0.5% glucose and 25 µg/ml chloramphenicol. Several individual clones were inoculated to SB medium supplemented with 25 µg/ml chloramphenicol, grown overnight at 37 °C and 250 rpm, and verified by DNA sequencing from miniprep DNA. The proven clone was inoculated to 100 ml of SB medium supplemented with 0.5% glucose and 25 µg/ml chloramphenicol and incubated overnight at 30 °C and 300 rpm. The pre-culture was diluted to OD600 0.05 in 500 ml of SB medium supplemented with 0.05% glucose and 25 µg/ml chloramphenicol and incubated at 37 °C and 250 rpm until OD600 reached 0.6–0.8. The culture was induced with 200 µM IPTG, and expression was carried out overnight at 26 °C and 250 rpm. The cells were harvested at 8000 g for 10 min at 4 °C (JA-10 rotor, Beckman Coulter, USA). The pellet was suspended in 50 ml of lysis buffer composed of 1 × PBS, 10 mM imidazole, 1 mM MgCl_2_, and 250 U Pierce Nuclease (Thermo Scientific, USA), pH 7.4. The cells underwent three freeze–thaw cycles followed by metal affinity purification with 2 ml of HisPur Ni–NTA Resin (Thermo Scientific, USA). The antibody was eluted from the column with the elution buffer (1 × PBS, 333 mM imidazole, pH 7.4) following the resin wash with five column volumes (CV) of the wash buffer (1 × PBS, 25 mM imidazole, pH 7.4). Ten 0.5-ml fractions were collected during the elution step. The 10-µl aliquot of elution fractions was mixed in a 1:1 ratio with 2 × Laemmli sample buffer (Bio-Rad, USA) containing 5% 2-mercaptoethanol and boiled for 5 min at 95 °C. The reduced samples were transferred to the wells of 4–15% Mini-PROTEAN TGX Precast Protein Gel (Bio-Rad, USA) and run at 100 V. The gel was stained for 2 h on gentle shaking using ReadyBlue Gel Stain (Sigma-Aldrich, USA) and washed with Milli-Q water (Merck, USA). The selected elution fractions were collected and concentrated using Vivaspin 4 Turbo 10 K MWCO Ultrafiltration Unit (Sartorius, Germany) followed by buffer exchange to 1 × PBS, pH 7.4, with NAP-10 desalting column (Cytiva, USA). Size exclusion chromatography (SEC) was performed using a Superdex 75 10/300 GL column (Cytiva, USA) with a flow rate of 0.5 ml/min and 1 × PBS, pH 7.4, as the buffer. Selected SEC fractions were collected and subsequently analysed by SDS-PAGE, following the procedure described above. Targeted fractions were combined and concentrated to 1 ml.

### Expression and purification of IgG antibody

All primers and gBlock gene fragments were ordered from IDT (Integrated DNA Technologies, USA). To generate desirable constructs, we introduced *XhoI* and *AflII* restriction sites to the heavy and light chain genes to enable cloning into the constitutive mammalian expression vector pcDNA3.4. By overlap extension PCR we connected the light and heavy chain genes with the SpyCatcher003 gene and similarly introduced *XhoI* and *AflII* restriction sites prior to cloning into pcDNA3.4 vector. Sequences and plasmid preparation for pcDNA3.4-LC and pcDNA3.4-HC antibody constructs are described in detail in Supplementary Information. The obtained plasmids were transformed into electrocompetent XL1-Blue *E. coli* cells, and transformant cells were grown on LA-agar plates supplemented with 0.5% glucose and 100 µg/ml ampicillin. Then, colonies were inoculated into a 3 ml SB medium supplemented with 100 µg/ml ampicillin and incubated overnight at 30 °C and 300 rpm. The successful insertion to pcDNA3.4 was proven by sequencing with primers AKV_020 and AKV_021. The PureLink HiPure Plasmid Maxiprep Kit (Thermo Scientific, USA) was used to obtain enough plasmid DNA needed for transfection.

The ExpiCHO-S cells (Gibco, USA) were cultured according to the manufacturer’s instructions. IgG antibody constructs were expressed in a 35-ml culture volume by co-transfecting ExpiCHO cells with pcDNA3.4-LC (25 µg DNA) and pcDNA3.4-HC (12.5 µg DNA) according to the manufacturer’s Max Titer protocol. On the 14th day post-transfection, the supernatant was collected by centrifugation at 3000 g for 20 min at 4 °C. Supernatant was filtered through 0.22 µm filter unit, and used to purify IgG antibodies with 1 ml HiTrap Protein A HP column on ÄKTA Explorer system (Cytiva, USA) following the manufacturer’s instructions and a flow rate of 1 ml/min. The antibodies were eluted from the column with 0.1 M citric acid, pH 3.0, and immediately neutralized with 1 M Tris–HCl, pH 9.0. The targeted fractions were combined and concentrated to 1 ml with Vivaspin 500 50 K MWCO centrifugal concentrator (Sartorius, Germany). Protein purity was confirmed by sodium SDS-PAGE analysis. Precision Plus Protein™ Dual Color standard was used as a marker (Bio-Rad, USA).

### Differential scanning fluorometry (DSF)

Thermal unfolding profiles of purified scFv-SpyCatcher003 (4 µM) and IgG-SpyCatcher003 (4 µM) antibodies were determined with 8 × SYPRO Orange dye (Sigma, S5692) and CFX96 Touch RT-PCR System (Bio-Rad, USA). The fluorescence intensity of SYPRO Orange was monitored while increasing the temperature from 4 to 98 °C with a 0.5 °C increment every 10 s. The melting temperature (Tm) was determined by Boltzmann sigmoidal curve fitting.

### SpyTag003-peptide labelling and purification

The SpyTag003-polyK peptide RGVPHIVMVDAYKRYKGGGGSKKKKKKKKKKKKKKKKKKKK (GenScript, USA) was dissolved in Milli-Q water to 1 mg/ml, and 0.5 mg of the peptide was labelled with 75 × molar excess of Eu(III)-N1-isothiocyanate (ITC) chelate (PerkinElmer, USA) over peptide. The reaction was carried out overnight in 50 mM carbonate buffer, pH 10.2, at RT with rotation. The Eu-labelled SpyTag003-polyK peptide was purified with HyPURITY C18 HPLC column 5 µm particle size on the UltiMate 3000 HPLC System. The targeted peak was collected and evaporated on the Savant SpeedVac SC210A concentrator using the Savant UVS400 vacuum system (Thermo Scientific, USA). Then, SpyTag003-polyK peptide was dissolved in 1 × TSA (50 mmol/l Tris–HCl, pH 7.75, 150 mmol/l NaCl, 0.5 g/l NaN_3_), and stored at 4 °C prior to coupling with antibodies.

### Coupling of SpyTag003-peptides with antibodies

The SpyTag003 peptide RGVPHIVMVDAYKRYK (10 µM) was mixed with scFv-SpyCatcher003 (15 µM) or IgG-SpyCatcher003 (25 µM) in 1 × PBS buffer, pH 7.4, and reactions were incubated overnight at 4 °C with rotation. The coupled scFv-SpyCatcher003/SpyTag003 and IgG-SpyCatcher003/SpyTag003 antibodies were purified with SEC. The targeted fractions were collected and concentrated with Vivaspin 4 Turbo 10 K MWCO ultrafiltration unit.

The Eu-labelled SpyTag003-polyK peptide (10 µM) was mixed with scFv-SpyCatcher003 (15 µM) or IgG-SpyCatcher003 (25 µM) in 1 × PBS buffer, pH 7.4, and reactions were incubated overnight at 4 °C with rotation. The coupled Eu-labelled scFv-SpyCatcher003/SpyTag003-polyK and Eu-labelled IgG-SpyCatcher003/SpyTag003-polyK were purified with SEC. The flow rate was 1 ml/min, and 0.4 ml fractions of the targeted peak were collected. Then, 1–10 µl of each fraction was diluted in DELFIA enhancement solution, incubated for 5 min at RT on slow shaking, and measured with Victor 1420 time-resolved plate fluorometer (PerkinElmer, USA). Fractions with the highest TRF signal were combined and concentrated using a Vivaspin 4 Turbo 10 K MWCO ultrafiltration unit. The absorbance at 280 nm was measured with a NanoDrop 1000 Spectrophotometer to calculate the labelling degree of the obtained antibodies.

### Labelling of control antibodies

The coupled scFv-SpyCatcher003/SpyTag003 (0.5 mg) and IgG-SpyCatcher003/SpyTag003 (0.5 mg) antibodies were labelled with 25-fold or 100-fold (scFv) as well as 50-fold, 150-fold and 300-fold (IgG) molar excess of Eu(III)-N1-ITC chelate. The labelling reaction was carried out overnight in 50 mM carbonate buffer, pH 9.5. The Eu-labelled antibodies were purified with SEC, and fractions with the highest TRF signal were determined as described above. Selected fractions were pooled together and concentrated. The absorbance at 280 nm was measured to calculate the labelling degree.

### TRFIA with scFv antibody

The assay buffer, wash buffer, DELFIA enhancement solution and streptavidin-coated plates were from Kaivogen Oy, Finland. The biotinylated anti-cardiac troponin I 4C2 Fab (100 ng/well) in 200 µl of the assay buffer was added to the pre-washed streptavidin-coated strips, and the plate was incubated for 1 h at RT on slow shaking. Then, the plate was washed four times, and 50–800 ng/l of human cardiac troponin I-T-C complex (HyTest Ltd, Finland) in 200 µl was added to the wells in triplicates. The plate was incubated for 1 h at RT on slow shaking followed by four washes. Then, Eu-labelled (25 ng/well; ø0.22 µm) 11N11 scFv antibody was added in 200 µl volume followed by 1 h incubation at RT on slow shaking. The plate was washed four times, and 200 µl of DELFIA enhancement solution (acidic chelating detergent solution) was added to each well to develop the fluorescence signal. The plate was incubated for 10 min at RT on slow shaking, and TRF signal was measured with Victor 1420.

### TRFIA with IgG antibody

The biotinylated anti-cardiac troponin I 4C2 Fab (50 ng/well) and 19C7 Fab (50 ng/well) in 200 µl of the assay buffer were added to the pre-washed streptavidin-coated strips, and the plate was incubated for 1 h at RT on slow shaking. The plate was washed four times, and 1–16 ng/l of human cardiac troponin I-T-C complex in 100 µl was added to the wells in triplicates. Also, Eu-labelled 11N11 IgG antibody (20 ng/well; ø0.22 µm) was added in 100 µl volume to the wells. The plate was incubated for 25 min at RT on slow shaking followed by four washes. The DELFIA enhancement solution (200 µl/well) was added to the plate for 5 min incubation at RT and slow shaking. The TRF signal was measured with Victor 1420.

### Data analysis

All immunoassay data were collected from triplicates and analyzed with GraphPad Prism 8 software (Dotmatics, USA). Linear regression analysis was performed to estimate the limit of detection. The theoretical isoelectric point (pI) for proteins and peptides was calculated with the ProtParam tool (https://www.expasy.org/resources/protparam) according to the predicted amino acid sequences.

### Supplementary Information

Supplementary Information.

## Data Availability

The authors declare that the data supporting the findings of this study are available within the paper and its supplementary information files. Primary data are available from the corresponding authors on reasonable request.

## Abbreviations

cTnI: Cardiac troponin I
DELFIA: Dissociation-enhanced lanthanide fluoro immunoassay
DoL: Degree of labelling
DSF: Differential scanning fluorometry
HPLC: High-performance liquid chromatography
IEF: Isoelectric focusing
IgG: Immunoglobulin G (full-length antibody)
ITC: Isothiocyanate
Ln: Lanthanide
LoD: Limit of detection
PBS: Phosphate-buffered saline
polyK: Polylysine
scFv: Single-chain variable fragment
SEC: Size exclusion chromatography
T_m_: Melting temperature
TSA: Tris-buffered saline with azide
TRFIA: Time-resolved fluorescence immunoassay
