# Supplementary material for: Controlled labelling of tracer antibodies for time-resolved fluorescence-based immunoassays
Source: Sci Rep. 2024 Aug 5;14:18113. doi: 10.1038/s41598-024-69294-7 (PMC11300886; doi:10.1038/s41598-024-69294-7)
Supplement: Supplementary file 1 — Supplementary Information. [file 41598_2024_69294_MOESM1_ESM.pdf]

Supplementary information

Title

Controlled labelling of tracer antibodies for time-resolved fluorescence-based immunoassays

Authors and affiliations

Anastasiia Kushnarova-Vakal[1,2,*], Rami Aalto[1,3], Tuomas Huovinen[1], Saara Wittfooth[1], and Urpo Lamminmäki[1,2,*]

[1] Department of Life Technologies, University of Turku, Turku 20520, Finland

[2] InFLAMES Research Flagship, University of Turku, 20014 Turku, Finland

[3] Tyks Laboratories, Clinical Chemistry, Turku University Hospital, Turku 20521, Finland

* Corresponding authors: amkuva@utu.fi; urplammi@utu.fi

Supplementary Table 1. Primers used in the study.

**AKV_006**	5’–AACTTAAGTCCCTACCGGTTAGTAATGAGTTTGA-3’
**AKV_007**	5’-AACTCGAGGATCCTCTAGAGTCCGGAGGC-3’
**AKV_010**	5'-AAATGCTCGAGATGTACAGGATGCAACTCCTGTCTTGCATT-3'
**AKV_011**	5'-AACTTAAGTTATCAGCACTCGTTTCTGTTGAAGCTCTTCACGAT-3'
**AKV_012**	5’–AACTTAAGTTATCACTTGCCGGGAGACAGGGACA-3'
**AKV_014**	5’-CTGCCACTTCCAGATGATCCGCACTCGTTTCTGTTGAAGCTCTTCACGAT-3’
**AKV_015**	5’-CTGCCACTTCCAGATGATCCCTTGCCGGGAGACAGGGACA-3'
**AKV_016**	5’-GCTTCAACAGAAACGAGTGCGGATCATCTGGAAGTGGCAGCGGCA-3’
**AKV_017**	5’-TGTCCCTGTCTCCCGGCAAGGGATCATCTGGAAGTGGCAGCGGCA–3’
**AKV_018**	5’–AACTTAAGTTATCAGGTGTGGGCGTCGCCCT-3’
**AKV_020**	5’–ATCGCCTGGAGACGCCATCCACGCT-3’
**AKV_021**	5’–CCACATAGCGTAAAAGGAGCAACATAG-3’
**TH404**	5’–AGCGGTGCTAGTGCCCACCTGCAGTCAAGCGTAACCACCTTATCAGGTTTATCAGG-3’
**TH405**	5’-TTCAAGCTTACTAATGGTGATGGTGATGATGTTGCAGGTGAGTATGAGCGTCACCTTCAGTTG-3’
**TH406**	5’-GCTTGACTGCAGGTGGGCACTAGCACCGCT-3’
**TH407**	5’-CACCTGCAACATCATCACCATCACCATTAGTAAGCTTG-3’

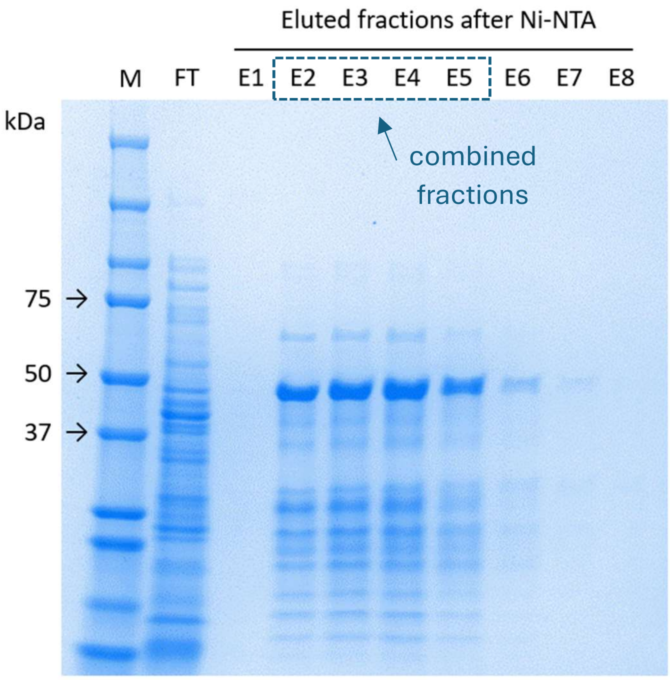

Supplementary Figure 1. SDS-PAGE analysis of the Ni-NTA purification of 11N11 scFv-SpyCatcher003. M, protein marker; FT, flowthrough; E1-E8, eluted fractions.

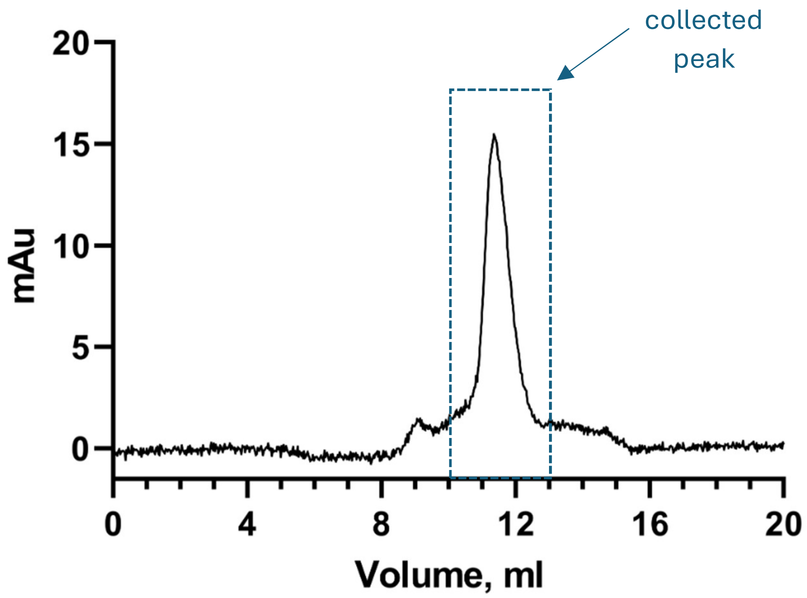

Supplementary Figure 2. Size-exclusion chromatography profile of the Ni-NTA purified 11N11 scFv-SpyCatcher003 obtained with a Superdex 75 Increase 10/300 GL column. mAU, milliarbitrary units.

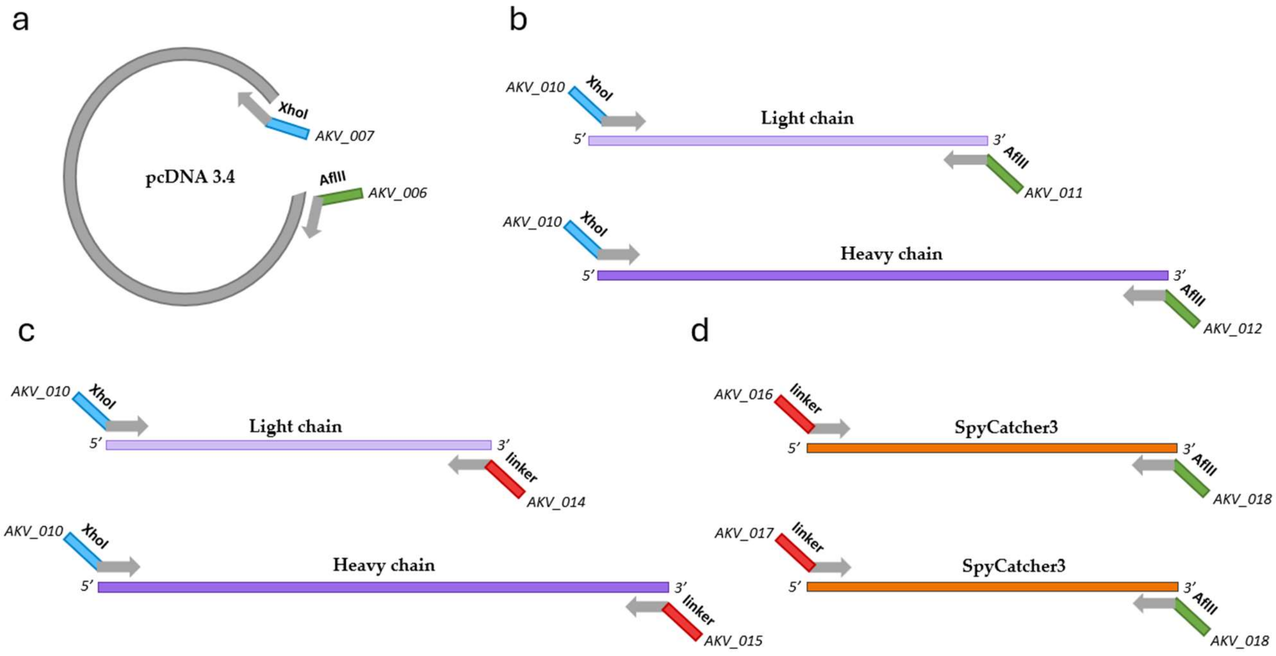

Supplementary Figure 3. OE-PCR details to get full-length antibodies with SpyCatcher003 on the C-terminus of heavy or/and light chain. To introduce *XhoI* and *AflII* restriction sites, pcDNA3.4 vector (**a**) was amplified with primers AKV_006 and AKV_007, while light and heavy chains of 11N11 IgG (**b**) were amplified with primers AKV_010, AKV_011 and AKV_012. Light and heavy chains were also amplified with AKV_010, AKV_014, and AKV_015 primers (**c**) to introduce the *XhoI* restriction site and overlapping region with SpyCatcher003, while SpyCatcher003 was amplified with AKV_016, AKV_017, and AKV_018 primers (**d**) to introduce *AflII* restriction site and overlapping region before the overlap extension PCR.

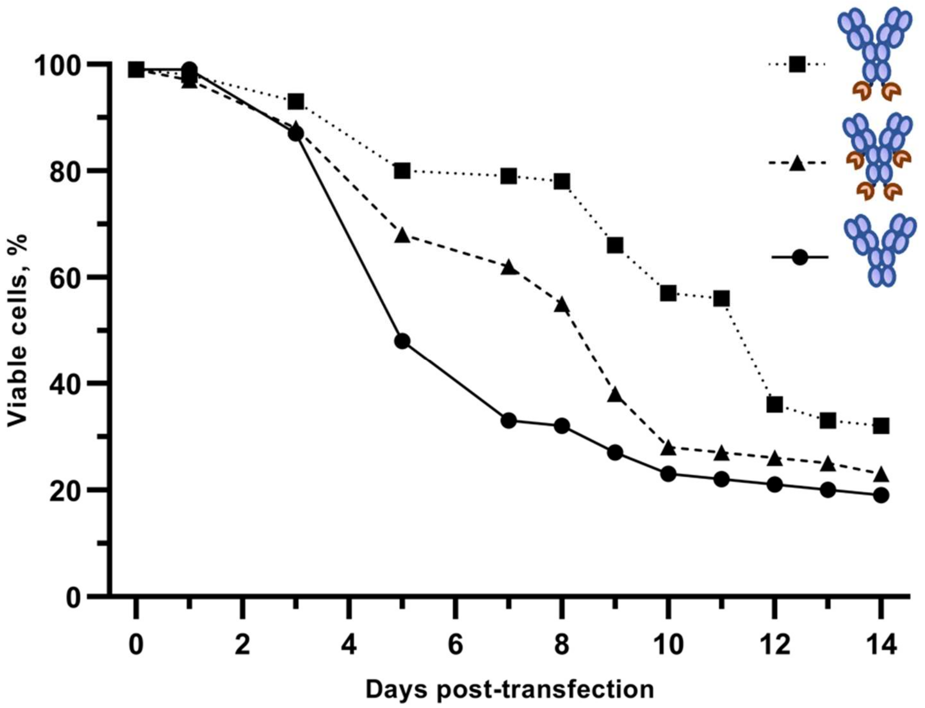

Supplementary Figure 4. Cell viability monitoring for 14 days post-transfection while transient expression of 11N11 IgG and IgG-SpyCatcher003 antibodies in ExpiCHO cells.

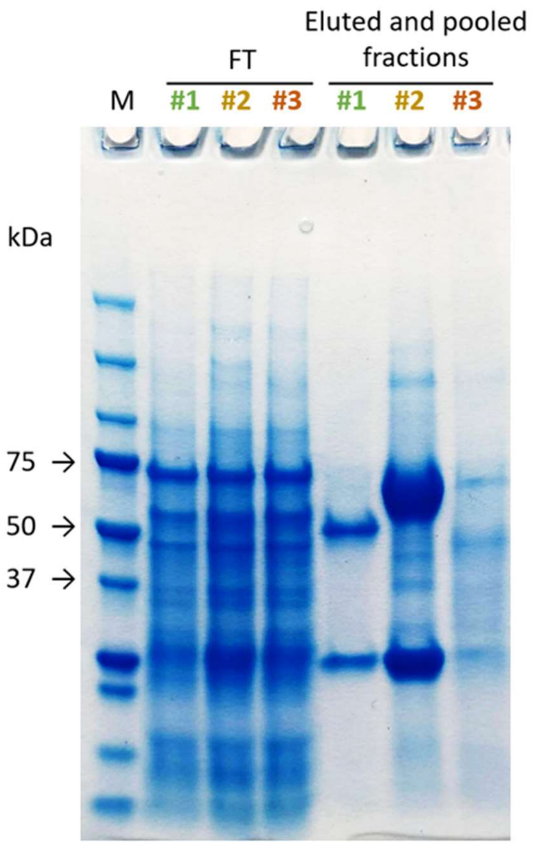

Supplementary Figure 5. SDS-PAGE analysis of the protein A purification of 11N11 IgG (**#1**) and 11N11 IgG-SpyCatcher003 (**#2**, SpyCatcher003 fused to the heavy chain; **#3**, SpyCatcher003 fused to both heavy and light chains). M, protein marker; FT, flowthrough.

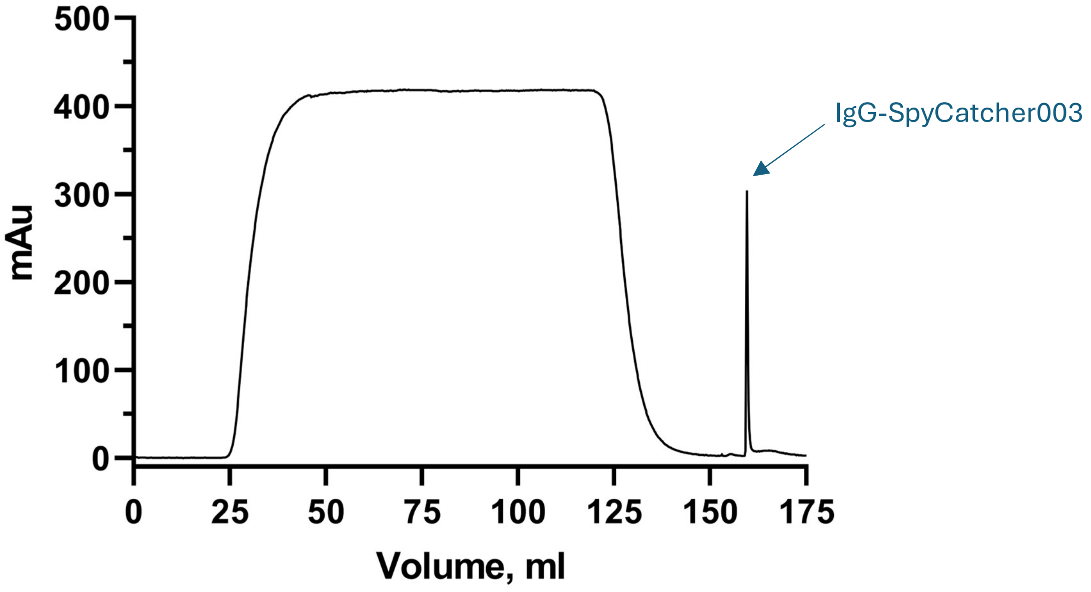

Supplementary Figure 6. Chromatogram of the 11N11 IgG-SpyCatcher003 purification using HiTrap Protein A HP antibody purification column.

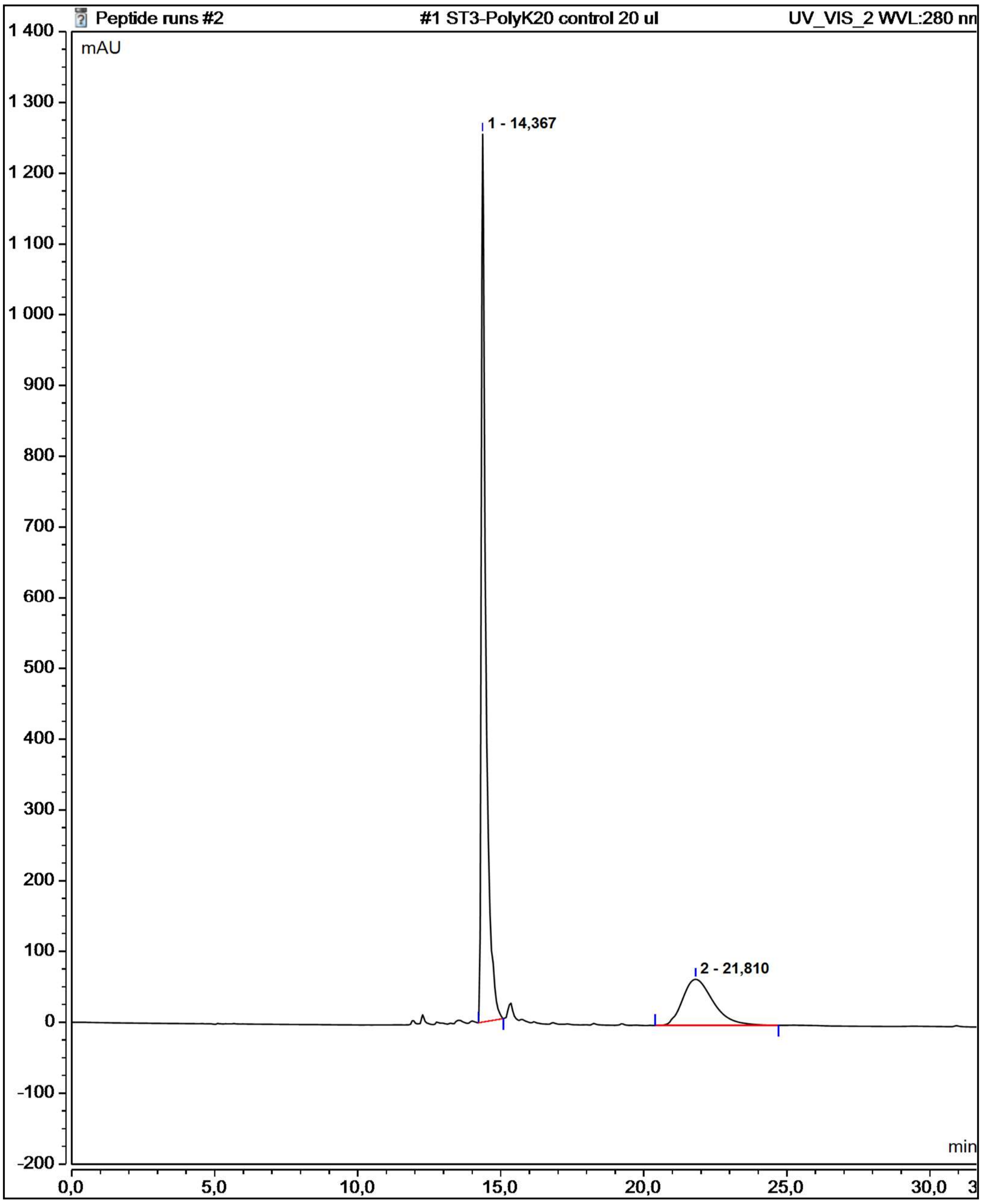

Integration Results						
No.	Peak name	Retention Time, min	Area, mAU*min	Height, mAu	Relative Area, %	Relative Height, %
1	Eu-chelate	14,367	229,779	1255,092	73,90	95,07
2	Eu-SpyT3-PolyK	21,810	81,172	65,038	26,10	4,93
**Total:**			**310,951**	**1320,130**	**100,00**	**100,00**

Supplementary Figure 7. Full HPLC profile and integration of chromatographic peaks for Eu-labelled SpyTag003-polyK$_{20}$ peptide.

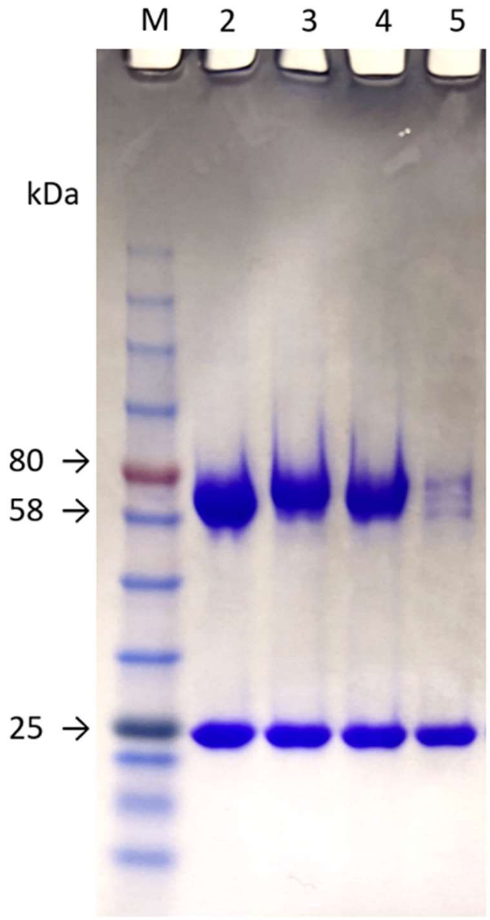

Supplementary Figure 8. SDS-PAGE analysis of the 11N11 IgG-SpyCatcher003 before and after complexation with SpyTag003-containing peptides. M, protein marker; lane 2, IgG-SpyCatcher003; lane 3, IgG-SpyCatcher003/SpyTag003; lane 4, IgG-SpyCatcher003/SpyTag003-biotin; lane 5, IgG-SpyCatcher003/SpyTag003-polyK_{20}.

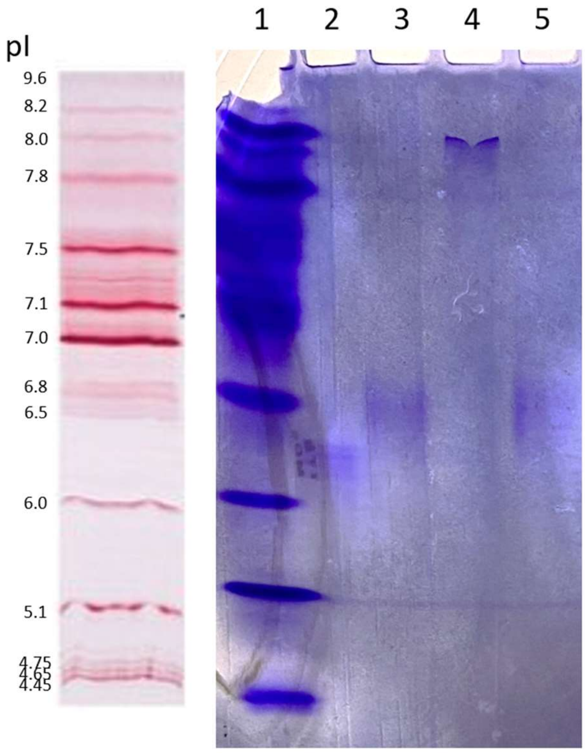

Supplementary Figure 9. IEF gel electrophoresis of 11N11 IgG-SpyCatcher003 before and after complexation with SpyTag003-containing peptides. Lane 1, IEF Standards; lane 2, IgG-SpyCatcher003; lane 3, IgG-SpyCatcher003/SpyTag003; lane 4, IgG-SpyCatcher003/SpyTag003-polyK$_{20}$; lane 5, IgG-SpyCatcher003/SpyTag003-biotin.

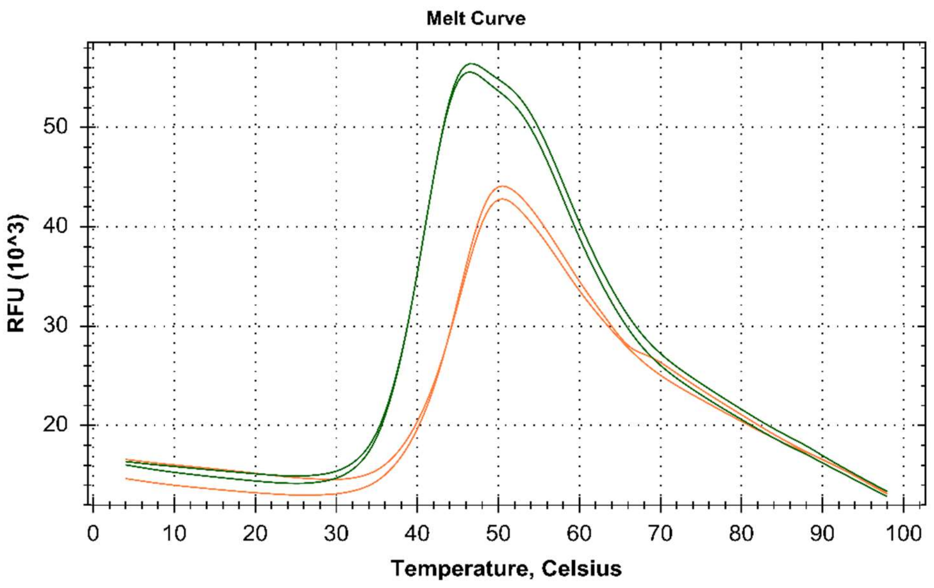

Supplementary Figure 10. Differential scanning fluorimetry (DSF) profiles of the 11N11 scFv-SpyCatcher003 (green) and 11N11 scFv-SpyCatcher003/SpyTag003 (orange) at 4 μM concentration in 1x PBS, pH 7.4 run in duplicates. SYPRO Orange (8x) was used as a reporter. The plot for fluorescence versus temperature dependence.

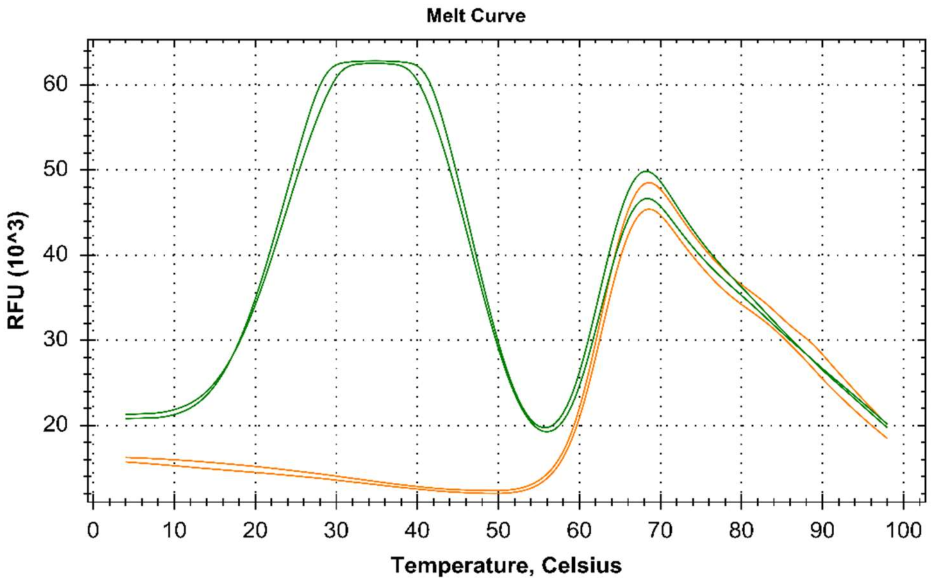

Supplementary Figure 11. Differential scanning fluorimetry (DSF) profiles of the 11N11 IgG-SpyCatcher003 (green) and 11N11 IgG-SpyCatcher003/SpyTag003 (orange) at 4 μM concentration in 1x PBS, pH 7.4, run in duplicates. SYPRO Orange (8x) was used as a reporter. The plot for fluorescence versus temperature dependence.

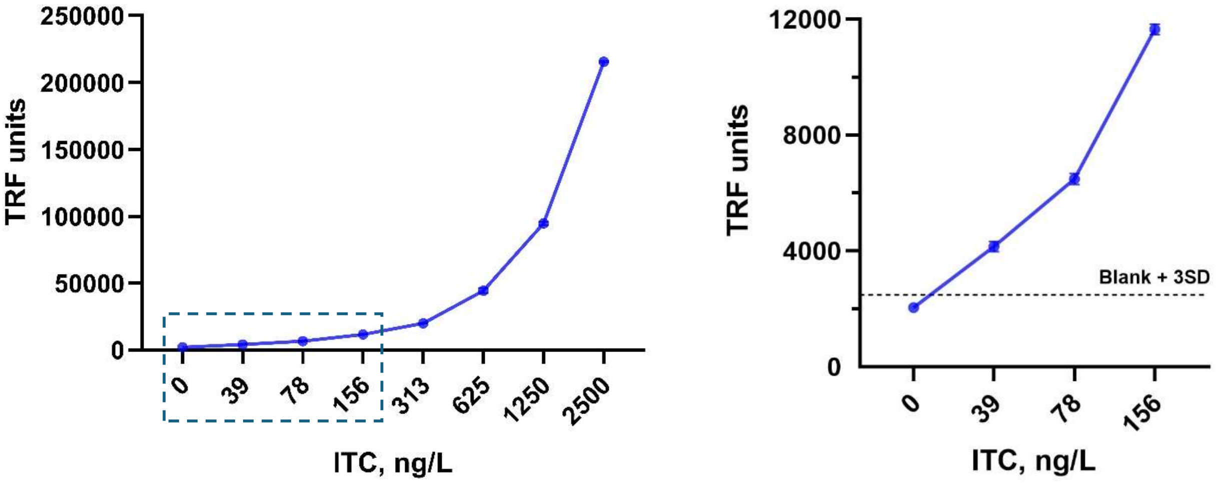

Supplementary Figure 12. The immunoassay dynamic range determination in response to increasing the ITC complex concentration. The data represents mean ± SD (n = 3). As a tracer, site-specifically labelled 11N11 IgG-SpyCatcher003/SpyTag003-polyK$_{20}$ antibody was used.
